# Effects of Modified Atmosphere Packaging with Different Gas Ratios on the Quality Changes of Golden Pompano (*Trachinotus ovatus)* Fillets during Superchilling Storage

**DOI:** 10.3390/foods11131943

**Published:** 2022-06-29

**Authors:** Xiaofan Zhang, Chuang Pan, Shengjun Chen, Yong Xue, Yueqi Wang, Yanyan Wu

**Affiliations:** 1 College of Food Science and Engineering, Ocean University of China, Qingdao 266003, China; zhangxiaofan19@sina.com (X.Z.); xueyong@ouc.edu.cn (Y.X.); 2 Sanya Tropical Fisheries Research Institute, Sanya 572000, China; wangyueqi@scsfri.ac.cn; 3 Key Laboratory of Aquatic Product Processing, Ministry of Agriculture and Rural Affairs, National R&D Centre for Aquatic Product Processing Technology, South China Sea Fisheries Research Institute, Chinese Academy of Fishery Sciences, Guangzhou 510300, China; wuyygd@163.com; 4 Collaborative Innovation Center of Seafood Deep Processing, Dalian Polytechnic University, Dalian 116034, China

**Keywords:** *Trachinotus ovatus*, golden pompano, modified atmosphere packaging, superchilling storage, physicochemical properties, principal component analysis

## Abstract

The quality changes of golden pompano fillets in air packaging (AP) and modified atmosphere packaging (MAP) with 30% CO_2_/70% N_2_, 50% CO_2_/50% N_2_, and 70% CO_2_/30% N_2_ were evaluated under superchilling (−3 °C). The results showed that the whiteness of fillets decreased during storage. The rate of pH increase of MAP was significantly slower than in AP groups, in which MAP with 70% CO_2_/30% N_2_ effectively suppressed the PH. Interestingly, the hardness decreased on day five following the treatments, followed by a relatively stationary trend. MAP could greatly suppress the increase of total volatile basic nitrogen (TVB-N) contents of fillets compared to fillets packed in AP. All MAP groups of fillets maintained first-grade freshness throughout storage, while the AP samples decreased to second-grade freshness on about the 25th day. MAP with 70% CO_2_/30% N_2_ and MAP with 50% CO_2_/50% N_2_ had the best results in inhibiting protein degeneration and explanation. Unexpectedly, drip loss of fillets in MAP far exceeded the AP group during storage, which causes sensory discomfort. Anaerobic plate count (APC) of fillets in AP exceeded the consumption limit of 6.7 log CFU/g on day 26 (6.75 log CFU/g on the 26th day), whereas the MAP was still microbiologically acceptable after 30 days of storage (6.43, 6.41, 6.22 log CFU/g, respectively). Considering physicochemical and microbiological parameters, the shelf life of fillets packed in AP was 25 days. MAP treatments could prolong the shelf life of fillets by ~4–5 days compared to AP. Overall, MAP with 70% CO_2_/30% N_2_ gas ratio was best for inhibiting the quality deterioration of fillets. Furthermore, principal component analysis (PCA) was performed to evaluate the critical indicators of quality deterioration of the fillets. Two principal components were determined by dimensionality reduction, in which the contribution of the first principal component was centrifugal loss > hardness > TVB-N > APC > CO_2_ solubility > TBARs > drip loss > pH, which mainly reflected the degree of microbial proliferation, protein hydrolysis, and oxidation. The contribution of the second principal component was pH > TBRAs > drip loss > APC > CO_2_ solubility > TVB-N > hardness > centrifugal loss, indicating a high correlation between lipid oxidation and microbial proliferation index.

## 1. Introduction

Golden pompano (*Trachinotus ovatus*) is a marine aquaculture economic fish of the genus *Trachinotus* in the family *Carangidae*, it has white, delicate flesh a delicious taste and is rich in protein and polyunsaturated fatty acids [1]. At present, golden pompano is mainly sold fresh, frozen or refrigerated, which is susceptible to protein, lipid oxidation and other spoilage phenomena during storage, resulting in short shelf life and reducing the economic value of golden pompano products [2].

Most aquatic products use low-temperature preservation, but traditional refrigeration (4 °C) shelf life is short. Moreover, frozen (−18~−20 °C) processing can easily cause protein denaturation, lipid oxidation and drip loss in the thawing process of aquatic products. However, superchilling is a temperature between refrigeration and freezing storage methods; the food temperature is usually controlled at 1–2 °C below freezing point [3]. Most microbial activities, protein deterioration and lipid oxidation are inhibited, moreover, the drip loss of aquatic products was at a lower level until the end of superchilling storage [4]. Compared with refrigeration, superchilling storage can effectively extend the shelf life of fillets by 1.5 to 4 times [5]. 

A previous study showed that superchilling (−3 °C) storage can effectively reduce microbial growth, lipid oxidation and proteolytic degradation in common carp surimi [6]. Mi et al. [4] reported that lower drip loss and higher water holding capacity under superchilling (−2 °C) storage were observed compared to frozen (−18 °C) storage and the shelf life of superchilled grass carp was 21 days. Liu et al. [7] also found that superchilling storage can inhibit the growth of microorganisms and retard the hydrolysis of proteins more effectively than at 0 °C in grass carp fillets. Superchilling preservation technology has many advantages in preserving food, but the temperature fluctuation, repeated freezing and thawing caused by the instrumentation or external environment can have adverse effects on the protein quality of the product [8,9]. Therefore, it is necessary to combine other preservation technologies to meet the market requirements for the freshness of aquatic products.

Modified atmosphere packaging (MAP) preservation technology has been widely used in aquatic preservation [10]. MAP refers to the use of high barrier performance packaging material to package food in which O_2_, CO_2_ and N_2_ gas are mixed into the packaging material to inhibit microbial growth [11], reduce the enzymatic reaction and slow down the rate of lipid oxidation [12]. It was illuminated that MAP significantly reduced the growth rate of pH, TVB-N and TBARs values and prolonged the shelf life of bream blocks [10]. Similar results were reported in a study of rainbow trout fillets [13]. The storage temperature can directly affect the deterioration rate of aquatic products and the combination of MAP and superchilling can significantly improve product quality [14]. Some researchers studied the preservation effect of MAP (60% CO_2_/40% N_2_) on fresh Atlantic salmon fillets at −2 °C and 4 °C. It was revealed that the quality of MAP salmon fillets deteriorated slowly at superchilling storage, which could effectively maintain the good quality of salmon fillets for 24 days [15]. It was also claimed that the APC and thermophilic bacteria were lower during storage at −0.7 °C compared to 4 °C in catfish, meanwhile, the TVB-N and TMA proved that the quality of MAP catfish could be better maintained in the −0.7 °C [16]. The packaging cost of MAP products is mainly determined by the pre-cooling conditions, sales environment and packaging materials. The first two belong to the one-time investment of the factory, and the cost is determined according to scale. Packaging materials mainly have gas and packaging bags, etc.; according to a rough calculation, a product packaging cost price is about 0.071~0.092 EURO, more than ordinary air packaging and vacuum packaging cost price is slightly higher, but gas-packaged products’ shelf life and fresh quality is better, so the sales price is higher than ordinary packaging but greatly enhances the market competitiveness of the product [17].

MAP combined with superchilling technology has been applied to many aquatic products. However, the exact gas ratio that is best for golden pompano storage has not been explored and our research will help enrich the market for packaged fillet products and contribute to improving the economic value of golden pompano or other fish crops. Therefore, the main objectives of this study were: (1) to investigate the effect of MAP with different gas ratios on physicochemical and microbial changes, such as pH, texture, water retention, CO_2_ solubility, whiteness, TBARs, TVB-N and APC of golden pompano fillets by using MAP combined with superchilling; (2) to evaluate the critical indicators of quality deterioration of the packaging fillets during storage by PCA.

## 2. Materials and Methods

### 2.1. Sample Preparation

**Figure 1:**
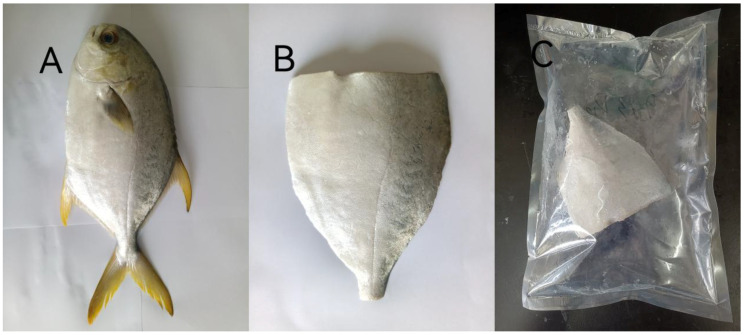
Images of golden pompano (**A**), fillet sample (**B**) and packaging method (**C**).

Fresh golden pompano (each fish weighing 500~600 g) (Figure 1A) were purchased from China Resources Vanguard supermarket in Guangzhou, China, accompanied by ice and returned to the laboratory within 5 min. Mucus was quickly washed off the surface with running water. Intact fillets (Figure 1B) of fish were removed from guts, black film, blood clots, etc. The surface water of the fillets was gently dried with kitchen paper and they were put into bags (Figure 1C) with high gas barrier performance (300 × 200) mm (Sinopharm Chemical Reagent Co., Ltd., Shanghai, China). Three different ratios of gas mixtures (30% CO_2_/70% N_2_; 50% CO_2_/50% N_2_; 70% CO_2_/30% N_2_) were selected using a gas conditioning preservation packaging machine (MAP-D400; Suzhou Senruy Co., Ltd., Suzhou, China) to create aerated packages named MAP1, MAP2 and MAP3 respectively. The control group used air packages named AP. Initial gas/fish volume ratio was 3:1 for all packages. 96 pieces of fillets were randomly divided into 4 groups for storage and 4 pieces were randomly taken from each group for parallel analysis during each experiment. All samples were stored together at −3 ± 0.5 °C in a precision low-temperature incubator (IN612C; Yamato Science Chongqing Co., Ltd., Chongqing, China).

The samples were taken at 0, 5, 10, 15, 20, 25 and 30 days after storage and analyzed for changes in muscle hardness, elasticity, CO_2_ solubility, whiteness, APC, pH, TVB-N, TBARs and water retention capacity.

### 2.2. Water Retention Analysis of Golden Pompano Fillets

#### 2.2.1. Determination of Drip Loss

Drip loss of fillets was estimated according to the method of Liu and Liang [7]. The weight of the fillets was taken before they were packed and recorded as W_1_. After storage, the juice on the surface of the fillets was absorbed using kitchen paper. The fillets were then weighed and recorded as W_2_. Triplicate samples were used to calculate drip loss according to Equation (1).

(1) $$\mathrm{drip}\;\mathrm{loss}\left(\%\right)=\frac{\left(W_{1}-W_{2}\right)}{W_{1}}\times 100\%$$

#### 2.2.2. Determination of Cooking Loss

Cooking loss of fish fillets was estimated using the method of Delles and Xiong [18] with light modification. The fillets were steamed for three minutes in a pot drawer containing boiling water at 100 °C. They were then weighed after gently absorbing the surface juice of the fillets with kitchen paper and recorded as W_3_. Triplicate samples were used to calculate drip loss according to Equation (2).

(2) $$cooking\;loss\left(\%\right)=\frac{\left(W_{2}-W_{3}\right)}{W_{2}}\times 100\%$$

#### 2.2.3. Determination of Centrifugal Loss

Determination of centrifugal loss according used the method of Delles and Xiong [18] with modification. A suitable size of filter paper was weighed on a balance and recorded as W_4_. Fish samples of 2 g were then accurately weighed, wrapped with filter paper and placed in a centrifuge tube. The samples were then put it in a freezing centrifuge at 1500 r/min for 15 min. After centrifugation, the residual fillets were removed from the filter paper. The mass of the filter paper after centrifugation was recorded as W_5_. The calculation (3) of centrifugal loss is as follows:

(3) $$centrifugal\;loss\left(\%\right)=\frac{\left(W_{5}-W_{4}\right)}{W_{4}}\times 100\%$$

### 2.3. Physical Analysis of Golden Pompano Fillets

#### 2.3.1. Texture Profile Analysis 

Texture profile analysis (TPA) was carried out by a texture analyzer (CT3; Brookfield, IL, USA). Fish blocks of 4 × 2 × 1.5 cm in length, width and height were taken for TPA texture analysis. The samples were placed on the platform and two cycles of TPA experiments were performed with a 4 mm diameter TA44 cylindrical probe with a trigger point load of 5 g; the test target was a distance of 5.0 mm; the test speed was 0.5 mm/s. The TPA parameters, such as hardness (kg) and elasticity (mm), were obtained. Six parallel samples were measured for each group.

#### 2.3.2. Determination of pH and Whiteness

2.0 g of each fillet was homogenized in 20 mL of distilled water. The pH was measured using a digital pH meter (IS128; Yimai Shanghai, Shanghai, China) and this was repeated three times. 

The color of fillets was measured using a Chroma Meter (CR-400; Konica Minolta, Tokyo, Japan) with the CIE color system. The results were expressed as brightness value L*, red-green value a* and yellow-blue value b*, where L* refers to lightness (0 is black and 100 is white), a* indicates greenness (*a* < 0) or redness (*a* > 0) and b* measures blueness (*b* < 0) or yellowness (*b* > 0) of samples. The colorimeter was calibrated using an instrument-configured whiteboard according to the manufacturer’s instructions. The used illuminant for colour measurement was D65 combined with a standard 2° observer, port/viewing area of ⌀11 mm. Each sample was repeated six times. The calculation of whiteness was referred to as the method of Pathare, Opara and Al-Said [19], and the calculation (4) of whiteness is as follows:

(4) $$\mathrm{whiteness}=\sqrt{{\left(100-L^{*}\right)}^{2}+a^{*}{}^{2}+b^{*}{}^{2}}$$

#### 2.3.3. Determination of Solubility of CO_2_

As previously described, the solubility of CO_2_ in fillets was determined using the method of Jakobsen and Bertelsen [20] with slight modifications. Two Brinell flasks were connected with latex tubes. A mixture of 25 mL of 12% perchloric acid and 25 mL of ethanol was added to one flask. 30 mL of 0.05 mol/L Ba(OH)_2_ standard solution was added to the other flask. Then 20 g of the churned fish was transferred to the flask containing the mixture of perchloric acid and ethanol. CO_2_ was released from the sample and dissolved into Ba(OH)_2_ solution, and the flask system was kept connected for 18 h. The Ba(OH)_2_ solution was rinsed from the flask with 20 mL of distilled water, filtered and the filter was rinsed with 10 mL of distilled water. The collected filtrate was titrated with 0.1 mol/L of HCl standard solution and three drops of phenolphthalein indicator. The solubility of CO_2_ was calculated as mL CO_2_/g sample. Samples were performed in triplicate.

### 2.4. Microbiological and Chemical Analysis of Golden Pompano Fillets

#### 2.4.1. Determination of the Aerobic Plate Count (APC) 

As per Yin et al. [13], 5 g of fillets was mixed with 45 mL of sterile saline. The content was diluted sequentially and 1 mL of each homogenate was added to a sterile culture dish. Plate count agar medium was added and the plate rotated to mix the content. After 72 h of incubation at 37 °C, the APC number was counted. The data were recorded as colony-forming units (CFU), expressed as log CFU/g. The samples were performed in triplicate.

#### 2.4.2. Determination of Total Volatile Basic Nitrogen (TVB-N) Value

The TVB-N content of fillets was determined using a modified assay from Zhu et al. [16]. The 10 g samples were stirred and then 75 mL of distilled water was added to a reaction flask and mixed. The flask was then left to stand for 30 min before 10 mL of oxidase solution was added and the mixture was distilled using a Kjeldahl nitrogen analyzer (KDN-19A, Shanghai Qianjian Instrument Co., Ltd., Shanghai, China). The distillate was collected in a receiving flask to which 30 mL of 20 mg/mL boric acid solution and 3 drops of the mixed indicator were added, which was generated by dissolving 0.1 g methyl red and 0.1 g methylene blue into 100 mL of ethanol. Finally, the fractions were titrated with 0.1 mol/L hydrochloric acid standard solution. TVB-N values were expressed as mg/100 g of samples. Three replicate experiments were performed.

#### 2.4.3. Determination of Thiobarbituric Acid-Reactive Substances (TBARs)

This was carried out according to the method of Cheng, Sun, Pu, Wang and Chen, [21] with slight modifications. Briefly, 5 g of fish samples were weighed and 25 mL of 7.5% trichloroacetic acid (containing 0.1% EDTA) was added, homogenized, shaken and extracted for 30 min. The mixture was then centrifuged for 5 min, 5 mL of supernatant was taken, and 5 mL of 0.02 mol/LTBA solution was added and heated in a boiling water bath for 20 min. The solution was left to turn pink and then cooled under running water. 5 mL of chloroform was then added and the mixture was shaken. This was then centrifuged for 5 min and the absorbance of the supernatant at 532 nm was measured. Three replicate experiments were conducted.

### 2.5. Statistical Analysis

The experiment was repeated three or six times and plotted using Origin 2021. The data were analyzed by one-way ANOVA and analysis of the significance of differences (*p* < 0.05) was carried out using SPSS 26.0. All the data were fitted to a normal distribution and correlation and dimensionality reduction analysis of indicators were performed using principal component analysis.

## 3. Results and Discussion

### 3.1. Effect of Different Gas Ratios on Water Retention of Golden Pompano Fillets 

**Figure 2:**
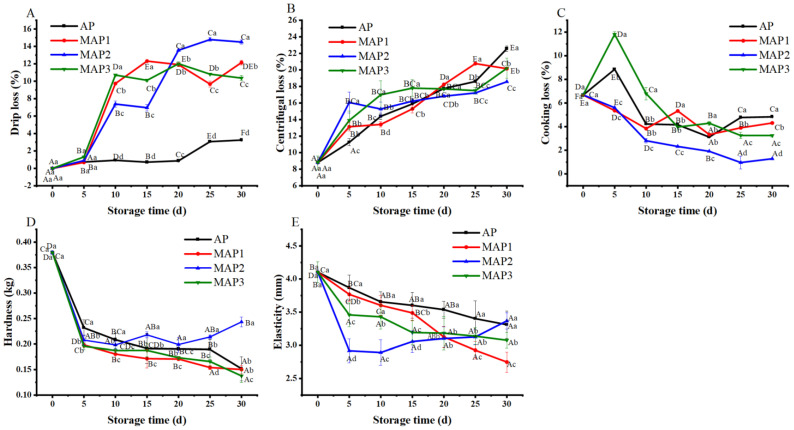
Effect of different gas components packaging on drip loss (**A**), centrifugal loss (**B**), cooking loss (**C**), hardness (**D**) and elasticity (**E**) of golden pompano fillets during storage (−3 °C). Packaging system: AP (control group), MAP1 (30% CO_2_/70% N_2_), MAP2 (50% CO_2_/50% N_2_), MAP3 (70% CO_2_/30% N_2_). Bars indicate the standard error. Different uppercase letters (A–F) indicate significant difference (*p* < 0.05) in means (n = 3) between display times within the same packaging system; different lowercase letters (a–d) indicate significant difference (*p* < 0.05) in means (n = 3) between packaging systems on the same day.

Drip loss will greatly affect consumers’ willingness to buy in that it might be seen to affect the juiciness, flavor, appearance and texture of fillets [7]. Figure 2A shows that drip loss of fillets in all groups increased during storage. AP had lower drip loss than all MAP groups, indicating that CO_2_ greatly affected the drip loss of fillets during superchilling storage. Generally, muscle water loss is mainly due to mechanical damage caused by ice crystallization and protein denaturation [22]. Meanwhile, CO_2_ is readily dissolved in aqueous and lipid phases, to produce carbonic acid that reduces the pH and contributes to protein denaturation [23]. Kimbuathong et al. [22] showed that the ambient group had lower drip loss with white shrimp than the MAP group. Several studies have reported that high CO_2_ gave higher drip loss and severe shrinkage of aquatic muscles due to reduced water holding capacity through protein decomposition [24,25]. Therefore, CO_2_ in the MAP significantly affected the degradation and oxidation of protein and broke the muscle tissue structure. Figure 2B shows that centrifugal loss of golden fillets increased continuously in all groups during storage. The centrifugal loss of AP increased faster than in the MAP groups. The maximum centrifugal loss of AP was 22.56% on the 30th day. However, the centrifugal loss of MAP2 was the lowest on the 30th day with 18.58%, which was the opposite of the results obtained from the drip loss of AP and MAP2. This was probably due to the excess water already precipitated from the fillets during superchilling storage, resulting in less water content in fillets. As shown in Figure 2C, the cooking loss of fillets in all groups showed a downward trend, among which the cooking loss of fillets in MAP2 was generally lower than in other groups.

### 3.2. Effect of Different Gas Ratios on Physicochemical Changes of Golden Pompano Fillets 

#### 3.2.1. Changes in Texture

Autolysis occurs immediately after the death of aquatic products due to the redistribution of water in the muscle and protein oxidation denaturation [26]. The changes in muscle hardness values of golden pompano fillets during storage are shown in Figure 2D. The initial hardness value of fresh fillets was 0.379 ± 0.003 kg. A sharp decrease in all groups was found in the first 5 days (*p* < 0.05), then the hardness value of fillets with AP decreased continuously with the extension of storage time and reached its lowest value of 0.152 ± 0.024 kg at the end of the 30th day. Decreased hardness is attributed to protease activity released from microorganisms and changes that take place within proteins [26]. Nevertheless, the hardness value of the MAP groups had no significant difference during storage (*p* < 0.05). This indicates that the MAP had an excellent preservation effect on the hardness value of fillets. In Figure 2E, the overall trend of the elasticity value of fillets was similar to hardness. The initial value of the elasticity of fillets was 4.10 ± 0.16 mm. When stored to the 30th day, the elastic value of fillets in the AP was high at 3.31 ± 0.20 mm, and the low elastic value in the MAP1 was 2.75 ± 0.15 mm. The decrease of texture in all groups was insignificant (*p* > 0.05) due to the better resistance to freezing of golden pompano [27], and the good antibacterial effect of MAP with CO_2_ during storage, which slowed down the growth of microorganisms in muscle. Meanwhile, the low temperature reduced the endogenous protease activities, thus inhibiting the oxidative denaturation of muscle. 

#### 3.2.2. Changes in Whiteness

**Figure 3:**
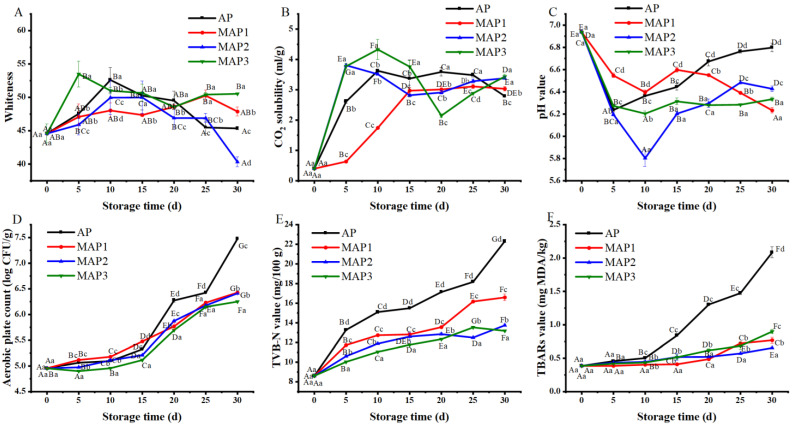
Effect of different gas components packaging on whiteness (**A**), CO_2_ solubility (**B**) and pH value (**C**), APC (**D**), TVB-N value (**E**) and TBARs value (**F**) of golden pompano fillets during storage (−3 °C). Packaging system: AP (control group), MAP1 (30% CO_2_/70% N_2_), MAP2 (50% CO_2_/50% N_2_), MAP3 (70% CO_2_/30% N_2_). Bars indicate the standard error. Different uppercase letters (A–G) indicate significant difference (*p* < 0.05) in means (n = 3) between display times within the same packaging system; different lowercase letters (a–d) indicate significant difference (*p* < 0.05) of means (n = 3) between packaging systems on the same day.

During the storage process, fillets will produce a lot of metabolic accumulation substances or oxidation of fish protein and lipids due to microorganisms and endogenous enzymes, resulting in unsatisfying colors such as yellow, red and dark [28]. As shown in Figure 3A, the whiteness of fillets in all groups showed a trend of rising and then falling, and the whiteness of fillets in MAP3 rose to the highest on the 5th day of storage. The reasons for whiteness variation may be due to the changes in muscle structure and water-binding state in the fillets caused by the superchilling temperature, which led to the strengthening of scattering intensity and the increase of whiteness. The water floated on the surface of the fillets after thawing due to the decrease in the water holding power of the fillets, which enhanced the reflection of light [29]. At the end of storage on the 30th day, all the groups differed significantly (*p* < 0.05). The MAP2 dropped to the lowest whiteness value, contrary to that, MAP2 had the highest drip loss during storage. It is demonstrated that the strength of muscle water-holding capacity affects the changes in color and brightness of fillets. Overall, the MAP1 with 30% CO_2_/70% N_2_ gas ratio had the slightest changes in whiteness value during storage, which maintained the best whiteness.

#### 3.2.3. Changes in CO_2_ Solubility

The pH and acid-base equilibrium were closed related to the solubility of CO_2_ in fillets. As shown in Figure 3B, the solubility of CO_2_ in all groups showed an increasing trend in the early stage (first 10 days) of storage, among which the AP, MAP1 and MAP2 showed a fast rate of increase. In contrast, the increase rate of CO_2_ solubility in the MAP1 group was slow and much lower than in the other three groups. In the middle and late storage stages (10–30 days), the increase of CO_2_ solubility in all groups tended to level off with little difference (*p* > 0.05), indicating that saturation was reached in the presence of CO_2_. Moreover, the solubility of CO_2_ was basically in the range of 2.82 ± 0.03~4.33 ± 0.34 mL/g, which was 87.22~90.05% higher than the initial CO_2_ solubility value. 

#### 3.2.4. Changes in pH Value

Generally speaking, the pH value of aquatic animals shows a decreasing trend and then increases after death. As shown in Figure 3C, the pH value of AP dropped to its lowest level on the 5th day of storage, then the pH value increased with storage time. The rate of pH increase of AP was significantly greater than MAP groups (*p* < 0.05). The initial decreases in pH might be related to the production of lactic acid and inorganic phosphate, while the increases during the later stages of storage may be due to the accumulation of ammonia and trimethylamine resulting from autolytic and microbial reactions [7,21]. The MAP groups had different degrees of decline in the first 10 days of storage, of which the MAP2 had the most significant decline (*p* < 0.05). This is probably due to the dissolution of some CO_2_ gas into the fillets or the difference in its material. The pH of the MAP groups started to rise slowly after 10 days, suggesting that MAP can better prevent microbial spoilage, which may lead to alkaline components, such as ammonia and trimethylamine [7]. Overall, the MAP3 with 70% CO_2_/30% N_2_ was more suitable for storage under superchilling (−3 °C). 

#### 3.2.5. Changes in TVB-N Value

TVB-N is the collective name of the protein decomposition of aquatic products in the storage process generated by ammonia, amines and other volatile alkaline nitrogenous substances [30]. As shown in Figure 3E, the TVB-N values of all groups increased and did not exceed 30 mg/100 g until the storage end. The fastest increase of TVB-N values could be seen in AP, followed by the MAP. The TVB-N values of the AP reached their highest level of 22.33 ± 0.16 mg/100 g on the 30th day of storage. This phenomenon might be due to formatted lactic acid dissolved in the filletswhich would result in a more remarkable pH change and cause a rise in TVB-N value [31]. However, the slowest increase was that of the MAP groups, and the highest TVB-N value in MAP1 was only 16.59 ± 0.34 mg/100 g, far below the AP. This is mainly because the higher CO_2_ content in MAP could suppress bacterial activity and delay chemical reactions [15]. According to the National Aquatic Industry Standard-Fresh and Frozen Pomfret (SC/T 3103-2010 standard), the content of the TVB-N does not exceed 18 mg/100 g for first-grade freshness and does not exceed 30 mg/100 g for second-grade freshness. All MAP groups of fillets maintained first-grade freshness throughout the storage, while the AP samples decreased to second-grade freshness on about the 25th day. In terms of TVB-N content, compared to the AP, MAP with superchilling storage could extend the shelf life of fillets by 5 days. The MAP3 with 70% CO_2_/30% N_2_ gas ratio had the best results, which were similar to the results of Lan, Xie, Zhu and Zhu [32], who found that the MAP could extend the shelf life of refrigerated pomfret by about 2 ~ 8 days and that the gas-conditioning ratio of 80% CO_2_/20% O_2_ was the best.

#### 3.2.6. Changes in TBARs Value

TBARs value is commonly used to determine the degree of fatty acid oxidation of aquatic products [29]. TBARs values indicate lipid oxidation in fillets (Figure 3F). The MAP can effectively inhibit the lipid oxidation of fillets and the trend of TBARs values in AP was similar to that of TVB-N during storage. It shows a slow increase followed by a rapid increase, while the TBARs values in the MAP increased slowly throughout the storage process. There were almost no significant changes in the TBARs value of all groups in the first 10 days (*p* > 0.05), indicating that the initial period of storage (first 10 days) was the induction period of fatty acid oxidative decay [26]. After 10 days of storage, TBARs values in AP increased rapidly due to the contact area of fatty acids with O_2_ promoting the lipid oxidation in fish through the destruction of muscle cells [33,34]. The MAP groups maintained a low growth rate in the late storage period (20~30 days), indicating that CO_2_ and N_2_ could effectively inhibit lipid oxidation in fillets. The best effect of inhibition of lipid oxidation was found in MAP3. An increase in CO_2_ tended to give lower TBARs values, possibly due to limited microbial growth and the release of a lipolytic enzyme [22]. 

### 3.3. Effect of Different Gas Ratios on APC of Golden Pompano Fillets 

Bacterial growth was one of the main factors causing the spoilage of aquatic products [35,36]. Bacteria can use the rich protein, glycogen and other nutrients in aquatic products to reproduce during storage, increasing APC [35]. As shown in Figure 3D, the APC of all groups showed slow growth in the first 15 days and rapid growth in later storage. In many countries, the microbial guideline for fish and fish products indicates that 6.7 log units represent the consumption limit. However, the APC number alone cannot be used as an absolute inspection standard [6]. According to this guideline, the shelf life of AP was less than about 26 days (6.75 log CFU/g at the 26 day), whereas the MAP was still microbiologically acceptable after 30 days of storage (6.43, 6.41, 6.22 log CFU/g, respectively). Esteves, Guerra and Anibal [37] noted that the APC number of the MAP was always lower than 7.0 log CFU/g in the fresh samples edible line. López-Caballero, Gonçalves and Nunes [38] found that MAP with 80% CO_2_/5% O_2_/15% N_2_ and 60% CO_2_/5% O_2_/35% N_2_ effectively inhibited microbial growth in the first 3 days. The MAP groups can extend shelf life by about 4 days in terms of the APC indicator. The MAP3 with 70% CO_2_/30% N_2_ had the lowest APC number. These results showed that higher CO_2_ tended to limit microbial growth. CO_2_ extended the lag phase, reduced the growth rate of microorganisms in their logarithmic phase and inhibited the activity of succinate dehydrogenase and malate dehydrogenase enzymes in the Krebs Cycle of microorganisms [39,40].

### 3.4. Principal Component Analysis of Quality Indicators of Golden Pompano Fillets 

#### 3.4.1. Pearson Correlation Analysis of Quality Indicators

As shown in Table 1, correlation analysis was used to investigate the correlation between CO_2_ solubility, pH, drip loss, centrifugal loss, hardness, APC, TVB-N and TBARs. The strength of the correlations was expressed using Pearson correlation coefficients. Specifically, the correlation coefficients between CO_2_ solubility, pH, drip loss, centrifugal loss, and hardness were −0.611, 0.369, 0.649 and −0.663. It showed significance at the 0.01 level, thus indicating a highly significant correlation with these physical indicators. As the CO_2_ solubility in fillets increased, the pH and water retention decreased, resulting in higher drip loss and centrifugal loss, lower hardness and deterioration of the quality of fillets. The correlation coefficient values in APC, TVB-N and TBARs were all greater than 0.800 and showed a significance at the level of 0.01. This proved that there was an extremely significant positive correlation with these indicators. With the enhancement of microbial and enzymatic decomposition, the content of TVB-N and TBARs of fillets showed a significant increasing trend. The growth of microorganisms during the storage of fillets had a significant effect on the chemical quality deterioration of fillets. The correlation coefficient of TVB-N and TBARs was 0.858, proving that protein decomposition and lipid oxidation of fillets affected each other to a great extent. The protein decomposition promoted lipid oxidation and vice versa. The correlation coefficients in centrifugal loss, APC, TVB-N and TBARs were all greater than 0.500, with a significance level of 0.01. It suggested that the decreased water retention of fillets is related to microorganisms, endogenous enzymes and oxidation actions. 

#### 3.4.2. Principal Component Analysis of Quality Indicators

The principal component analysis was conducted using SPSS 26.0 software. The characteristic roots, variance contribution rates and cumulative variance contribution rates of each principal component were obtained. As shown in Table 2, there were two principal components with characteristic roots greater than 1.000 and the sum of the contribution of the first and second principal components was 80.231%. The two principal components could reflect most of the information of the original variables. From Table 3, it can be seen that the eight indicators have a significant correlation and information overlap. Therefore, the indicators during storage were reduced from the initial eight to two principal components to achieve the purpose of dimensionality reduction. CO_2_ solubility, pH, drip loss, centrifugal loss, hardness, APC, TVB-N and TBARs all have high loadings on the first and second principal components, indicating that the two principal components can mainly reflect the information of these indicators. The score coefficients indicate the degree of influence of each indicator on the principal components, and the score coefficients can be used to linearly combine each variable to establish the functional relationships between the first principal component (*Y*_1_) and the second principal component (*Y*_2_). The indicators are CO_2_ solubility (*X*_1_), pH (*X*_2_), drip loss (*X*_3_), centrifugal loss (*X*_4_), hardness (*X*_5_), APC (*X*_6_), TVB-N (*X*_7_) and TBARs (*X*_8_). The score coefficient model for the eight variables was obtained from Table 3. Thus, the first principal component was extracted as Equation (5) and the second principal component score coefficient model was Equation (6).

(5) $$Y_{1}=0.448X_{1}-0.412X_{2}+0.403X_{3}+0.386X_{4}+0.332X_{5}+0.225X_{6}-0.193X_{7}+0.323X_{8}$$

(6) $$Y_{2}=-0.022X_{1}+0.214X_{2}+0.296X_{3}+0.332X_{4}-0.301X_{5}-0.335X_{6}+0.573X_{7}+0.480X_{8}$$

In the principal component loading matrix of Table 3, the absolute values of detection reflect the contribution to the principal components, and the magnitude of contribution in the first principal component was centrifugal loss > hardness > TVB-N > APC > CO_2_ solubility > TBARs > drip loss > pH. The absolute values of component coefficients of centrifugal loss rate, hardness, TVB-N and APC indicators were all greater than 0.800 (*p* < 0.01), and the absolute values of CO_2_ solubility and TBARs indicator were all greater than 0.600 (*p* < 0.01). This indicated that they were highly correlated with the first principal component. The first principal component mainly reflected the degree of microbial proliferation, protein hydrolysis and oxidation of fillets. The contribution of the second principal component was pH > TBARs > drip loss > APC > CO_2_ solubility > TVB-N > hardness > centrifugal loss. The absolute values of the component coefficients of pH, TVB-N, and TBARs were all greater than 0.600 (*p* < 0.01). This certainly reflected the high correlation between protein denaturation and lipid oxidation.

## 4. Conclusions

The quality changes of golden pompano fillets in AP and MAP with different gas ratios were evaluated at superchilling (−3 °C) storage. The results showed that MAP could effectively slow down the increase of pH, TVB-N value, TBARs value, hardness, elasticity, whiteness and APC of golden pompano fillets compared to AP. However, the water retention (drip loss, centrifugal loss, cooking loss) was poor in MAP. This phenomenon is not conducive to the sensory experience of packing fillets, making consumers lose interest. Pearson analysis showed that CO_2_ solubility was significant, positively correlated with drip loss and centrifugal loss and negatively correlated with pH and hardness (*p* < 0.01). Moreover, the coefficient values of the interrelationships among APC, TVB-N and TBARs were all greater than 0.800. This showed a significantly positive correlation (*p* < 0.01), which reflected the close relationship between microorganisms and deterioration of chemical quality. These quality indicators could be simplified into two principal components by PCA with the cumulative variance contributions of 54.847% and 80.231%, respectively, reflecting the original information better. A score coefficient model of the principal components was established, providing a reference for comprehensive quality evaluation of fillets. In conclusion, superchilling (−3 °C) combined with MAP could effectively maintain the quality of golden pompano fillets, and the gas ratio of 70% CO_2_/30% N_2_ has the best comprehensive freshness preservation effect.

## Figures and Tables

**Table 1:** Pearson correlation analysis of quality indicators in golden pompano fillets during storage.

Quality Indicator	CO_2_ Solubility	pH	Drip Loss	Centrifugal Loss	Hardness	APC	TVB-N	TBARs
CO_2_ solubility	1.000							
pH	−0.611 **	1.000						
Drip loss ^1^	0.369 **	−0.435 **	1.000					
Centrifugal loss ^2^	0.649 **	−0.370 **	0.555 **	1.000				
Hardness ^3^	−0.663 **	0.617 **	−0.513 **	−0.795 **	1.000			
APC ^4^	0.266 *	0.082	0.407 **	0.745 **	−0.495 **	1.000		
TVB-N ^5^	0.404 **	−0.063	0.150	0.720 **	−0.644 **	0.817 **	1.000	
TBARs ^6^	0.251 *	0.227 *	−0.068	0.579 **	−0.373 **	0.813 **	0.858 **	1.000

* indicates highly significant correlation at the *p* < 0.05 level. ** indicates highly significant correlation at the *p* < 0.01 level. ^1^ Defreeze lost water after storage. ^2^ Water holding of muscles. ^3^ Force necessary to obtain a given deformation.^4^ Anaerobic plate count. ^5^ Total volatile basic nitrogen. ^6^ Thiobarbituric acid-reactive substances.

**Table 2:** Principal component analysis of quality indicator in golden pompano fillets during storage.

Component ^1^	Initial Eigenvalueinitial Eigenvalue			Extract Square and Load		
	Latent Root	Variance Contribution/%	Accumulative Variance Contribution/%	Latent Root	Variance Contribution/%	Accumulative Variance Contribution/%
1	4.388	54.847	54.847	4.388	54.847	54.847
2	2.031	25.384	80.231	2.031	25.384	80.231
3	0.808	10.101	90.332			
4	0.350	4.381	94.713			
5	0.168	2.102	96.815			
6	0.132	1.652	98.467			
7	0.076	0.950	99.417			
8	0.047	0.583	100.00			

^1^ Comprehensive indicators formed by data conversion and dimensionality reduction for eight quality indicators.

**Table 3:** Loading matrix for principal component analysis of quality indicator in golden pompano fillets during storage.

Quality Indicator	Component 1 Coefficient	Coefficient of Component 1 Score	Component 2 Coefficient	Coefficient of Component 2 Score
CO_2_ solubility	0.941	0.448	−0.046	−0.022
pH	−0.864	−0.412	0.305	0.214
Drip loss ^1^	0.845	0.403	0.422	0.296
Centrifugal loss ^2^	0.808	0.386	0.459	0.322
Hardness ^3^	0.695	0.332	−0.429	−0.301
APC ^4^	0.534	0.255	−0.478	−0.335
TVB-N ^5^	−0.405	−0.193	0.816	0.573
TBARs ^6^	0.677	0.323	0.684	0.480

^1^ Defreeze lost water after storage. ^2^ Water holding of muscles. ^3^ Force necessary to obtain a given deformation. ^4^ Anaerobic plate count. ^5^ Total volatile basic nitrogen. ^6^ Thiobarbituric acid-reactive substances.

## Data Availability

The data presented in this study are available on request from the corresponding author. The data are not publicly available due to important national-level projects.
